# Workplace Violence Exposure and Job Burnout Among Korean Paramedics: The Moderating Roles of Family, Coworker, and Organizational Support

**DOI:** 10.3390/healthcare14121794

**Published:** 2026-06-22

**Authors:** Nayoon Lee, Daye Lee

**Affiliations:** 1 College of Nursing, Dong-A University, 32 Daesingongwon-ro, Seo-gu, Busan 49201, Republic of Korea; 095750@dau.ac.kr; 2 Graduate School, College of Nursing, Dong-A University, 32 Daesingongwon-ro, Seo-gu, Busan 49201, Republic of Korea

**Keywords:** paramedics, workplace violence exposure, job burnout, social support, family support, organizational support, coworker support

## Abstract

**Highlights:**

**What are the main findings?**

- Workplace violence exposure was positively associated with job burnout among Korean paramedics.
- Family support significantly moderated the relationship between workplace violence exposure and job burnout, indicating a buffering effect under high-stress conditions.
- Organizational support showed a negative but non-significant association with job burnout, whereas coworker support did not show significant effects.
- The present study provides evidence that family, coworker, and organizational support may play distinct roles in relation to burnout among paramedics.

**What are the implications of the main findings?**

- Organizational support systems may help reduce job burnout among paramedics exposed to workplace violence.
- Family-centered support strategies should be considered in interventions aimed at preventing burnout among paramedics.
- Mental health programs for paramedics may benefit from incorporating both organizational and family-based support approaches.

**Abstract:**

**Background/Objectives**: This study sought to investigate the relationship between workplace violence exposure and job burnout among Korean paramedics and the moderating roles of different sources of social support, including family, coworker, and organizational support, on this relationship. **Methods**: Participants were 175 paramedics working in B city, South Korea. Data were collected through an online survey conducted from 15 July to 30 July 2025. Workplace violence exposure, family support, coworker support, organizational support, and job burnout were assessed using validated self-report questionnaires. Descriptive statistics, correlation analyses, and three-step hierarchical regression analyses were performed using the SPSS program. **Results**: Workplace violence exposure was positively associated with job burnout among paramedics. Among the three sources of social support, organizational support was associated with lower levels of job burnout. Family support moderated the association between workplace violence exposure and job burnout, whereas the moderating effects of coworker support and organizational support were not statistically significant. **Conclusions**: The findings suggest that organizational support and family-based support strategies may be important resources for addressing job burnout among paramedics exposed to workplace violence. These findings may contribute to a better understanding of support mechanisms associated with job burnout among paramedics and inform future intervention development and organizational support strategies.

## 1. Introduction

Korean paramedics working in the 119 emergency medical service system are repeatedly exposed to crisis situations, including disaster scenes and life-threatening emergencies. Due to the nature of their work, they experience high levels of occupational stress and post-traumatic stress [1,2]. In addition, because their duties involve frequent interactions with patients and caregivers, they are also reported to experience substantial emotional labor [3]. High levels of occupational stress, post-traumatic stress, and emotional labor have been identified as major contributors to burnout, a state of psychological and emotional exhaustion. Similar trends have also been reported internationally, with previous systematic reviews showing that paramedics and ambulance personnel experience substantial levels of burnout, psychological distress, and occupational stress related to repeated exposure to traumatic events and emotionally demanding work environments [4,5,6].

Burnout not only negatively affects individual health by worsening physical health conditions and increasing psychological challenges, including depression, anxiety, and suicidal thoughts, but also adversely affects job performance and organizational functioning, including reduced work efficiency, decreased job satisfaction, and increased turnover intention [7,8]. Therefore, burnout among paramedics extends beyond an individual-level issue and may ultimately affect public safety, the quality of emergency medical services, and the stability of the emergency medical system [9]. Accordingly, active interventions aimed at reducing burnout among paramedics are urgently needed.

Among the various factors associated with burnout, workplace violence exposure has received considerable attention. Previous studies have investigated workplace violence exposure among nurses [10], care workers [11], social workers [12], and paramedics. Paramedics are frequently exposed to verbal abuse, threats, and physical assaults from patients and caregivers during duty [13]. In South Korea, a fatal incident involving a paramedic who died following workplace violence exposure occurred in 2018 [14], leading to increased social awareness and the implementation of legal and institutional measures to protect paramedics from workplace violence [15]. However, despite these efforts, workplace violence against paramedics has not decreased and has instead shown an increasing trend. This highlights the urgent need to identify protective factors that may alleviate burnout following workplace violence exposure, beyond merely focusing on post-incident responses.

Among the potential protective factors, social support has been regarded as particularly important. Although social support has consistently been identified as a protective factor against occupational stress and burnout, previous studies have often treated social support as a single construct. However, support from family, coworkers, and organizations may serve different functions and operate through distinct mechanisms. Understanding these source-specific roles is important because interventions and policies can be implemented at different levels, including family-based programs, peer-support initiatives, and organizational support systems. Therefore, examining social support according to its source may advance current understanding of source-specific support mechanisms and provide more practical and theoretically meaningful evidence for developing targeted interventions and policies to prevent job burnout among paramedics.

The present study is conceptually grounded in the Job Demands–Resources (JD-R) model [16] and the stress-buffering hypothesis [17]. According to the JD-R model, workplace violence exposure can be viewed as a job demand that depletes employees’ psychological resources and increases the risk of job burnout. In contrast, social support may function as a job resource that helps employees cope with occupational stressors. Furthermore, the stress-buffering hypothesis suggests that social support may mitigate the adverse effects of stressful experiences on psychological outcomes. Previous studies involving firefighters have suggested that the effects of social support may vary according to its source, with family, coworker, and organizational support demonstrating different associations with mental health outcomes [18,19]. These findings provide a theoretical basis for examining source-specific forms of social support rather than treating social support as a single construct. Workplace violence exposure among paramedics is a particularly relevant context in which these source-specific roles may emerge. Workplace violence typically occurs during emergency response activities conducted with coworkers, arises within an organizational context, and may continue to affect individuals after returning home, where family support becomes relevant. Therefore, family, coworker, and organizational support may each play distinct roles in the relationship between workplace violence exposure and job burnout. Examining these sources separately may provide a more comprehensive understanding of support mechanisms and help inform more targeted intervention strategies for paramedics. Based on these perspectives, the present study examined whether family support, coworker support, and organizational support moderate the relationship between workplace violence exposure and job burnout among paramedics. Accordingly, we hypothesized that each source of social support would buffer the adverse effects of workplace violence exposure on job burnout.

Previous studies have shown that social support enhances emotional stability and resilience and is negatively associated with job burnout. In particular, social support has been found to play a moderating role in the relationship between stress and burnout [19,20]. Song et al. [20] reported that social support weakened the association between job stress and burnout among 119 emergency medical technicians. Similarly, Ahn et al. [18] found that the moderating effects of social support differed according to its source among firefighters, suggesting that family, coworker, and organizational support may play distinct roles under stressful conditions. However, research examining this moderating effect remains limited.

Furthermore, the effects of social support may differ depending on its source. Previous studies involving firefighters have reported that the effects of social support vary according to its source, such as family, coworkers, and organizations [18,19]. For example, support from family, coworkers, and organizations has been shown to play different roles in psychological adaptation following traumatic experiences. Previous studies have reported that peer support may have a direct influence on positive psychological outcomes, whereas organizational support may contribute through different mechanisms and play an important role in long-term psychological adaptation [18,19]. These findings suggest that different sources of social support may operate through distinct mechanisms and exert varying influences under stressful conditions. If the role of social support in the association between workplace violence exposure and burnout among paramedics also differs by source, these findings could provide important evidence for developing more practical intervention programs and support strategies. Nevertheless, few studies have examined the moderating effects of different sources of social support on the relationship between workplace violence exposure and burnout among paramedics. Moreover, previous studies have often treated social support as a single construct, providing limited evidence regarding whether different sources of support operate through distinct mechanisms under stressful conditions. By separately examining family, coworker, and organizational support, the present study provides evidence regarding which sources of support may be most relevant in the association between workplace violence exposure and job burnout. Such evidence may help guide the development of more targeted intervention programs and support strategies for paramedics. Therefore, this study aimed to examine the moderating effects of family support, coworker support, and organizational support on the relationship between workplace violence exposure and job burnout among Korean paramedics.

## 2. Materials and Methods

### 2.1. Participants and Data Collection

This study was conducted to identify factors associated with job burnout among paramedics. Participants were convenience-sampled from paramedics working in B city, South Korea, who were actively engaged in field emergency response duties. Eligibility criteria included being aged 18 years or older, having at least 6 months of work experience as a paramedic, understanding the purpose of the study, and voluntarily agreeing to participate, regardless of prior workplace violence exposure.

Sample size estimation was performed using G*Power version 3.1.9.7 (Heinrich Heine University Düsseldorf, Düsseldorf, Germany). For multiple regression analysis, assuming a medium effect size (f^2^ = 0.15), a significance level of 0.05, a statistical power of 0.90, and 13 predictors, the minimum required sample size was estimated to be 162 participants. A total of 175 participants were included in the final analysis, exceeding the required sample size.

Data collection was conducted over approximately two weeks, from 15 July to 30 July 2025. Information regarding the study was distributed through the internal online bulletin board of the Busan Fire and Disaster Headquarters and the KakaoTalk channel operated by the Busan Firefighter Psychological Support Team. Paramedics who agreed to participate completed an online survey. Because the survey link was distributed through an internal online bulletin board and a KakaoTalk communication channel, the exact number of eligible paramedics who viewed the recruitment notice could not be determined. Therefore, the response rate could not be calculated.

The online questionnaire was administered through Naver Forms and accessed via a QR-code-based survey link. Prior to participation, respondents were presented with study information and were required to provide electronic informed consent before accessing the questionnaire. The study was approved by the Institutional Review Board (IRB) of Dong-A University (initial approval: IRB No. 2-1040709-AB-N-01-202504-HR-023-02, approved on 2 May 2025; amended approval: IRB No. 2-1040709-AB-N-01-202504-HR-023-06, approved on 3 July 2025).

### 2.2. Study Instruments

#### 2.2.1. Workplace Violence Exposure

Workplace violence exposure was measured using a tool originally developed based on the studies of Erickson, Williams-Evans, and Erickson and Williams-Evans [21] and Fernandes et al. [22], and later revised and supplemented by Yoon [23]. The instrument consisted of 16 items assessing three types of workplace violence: verbal violence (4 items), physical threats (5 items), and physical violence (7 items). Verbal violence was assessed based on weekly frequency, physical threats based on monthly frequency, and physical violence based on yearly frequency. Each item was rated on a 5-point frequency-based scale ranging from 1 (“less than once”) to 5 (“three times or more”), with higher scores indicating greater exposure to workplace violence. The overall reliability of the instrument was reported as 0.86 in the original study, and Cronbach’s α in the present study was 0.95.

#### 2.2.2. Job Burnout

Job burnout was measured using the validated Korean version of the Maslach Burnout Inventory–General Survey (MBI-GS) developed by Schaufeli et al. [24]. The instrument consists of 16 items across three subscales: exhaustion, cynicism, and professional efficacy. Responses are rated on a 6-point Likert scale ranging from 1 (“never”) to 6 (“every day”). In the present study, burnout was treated as an overall construct, and the total score was used in all analyses. Cronbach’s α for the overall scale was 0.88.

#### 2.2.3. Family Support and Coworker Support

Family support and coworker support were measured using a scale originally developed by Park [25] and later modified by Yoo [26]. The social support scale consists of 25 items, with responses rated on a 5-point Likert scale ranging from 1 (“strongly disagree”) to 5 (“strongly agree”). Higher scores indicate greater perceived social support. In Yoo’s study [26], Cronbach’s α values were 0.92 for emotional support, 0.91 for evaluative support, 0.92 for informational support, 0.87 for material support, and 0.97 for the overall scale. In the present study, Cronbach’s α for the overall scale was 0.97. This scale was originally developed and validated in Korea and has been widely used among Korean populations. Therefore, it was considered culturally appropriate for measuring family and coworker support in the present study.

#### 2.2.4. Organizational Support

Organizational support was measured using the Perceived Organizational Support scale originally developed by Eisenberger et al. [27]. Kim [28] modified the original 16-item instrument into a 15-item version using a 5-point Likert scale. A Likert summated scale approach was applied by calculating the mean scores of items classified under the same factor through factor analysis. In Kim’s study [28], the reliability coefficient was 0.88, whereas Cronbach’s α in the present study was 0.96. The Korean version of the Perceived Organizational Support scale was previously adapted and validated among Korean workers, supporting its cultural appropriateness and suitability for the present study.

### 2.3. Data Analysis

Statistical analyses were conducted using IBM SPSS Statistics version 29.0 (IBM Corp., Armonk, NY, USA). Descriptive statistics, including frequencies, percentages, means, and standard deviations, were used to summarize the general characteristics of the participants. The internal consistency of the measurement instruments was evaluated using Cronbach’s α coefficients.

Differences in workplace violence exposure and job burnout according to participants’ general characteristics were analyzed using independent *t*-tests and one-way analysis of variance (ANOVA), followed by Scheffé post hoc tests. Pearson’s correlation coefficients were calculated to examine relationships among workplace violence exposure, job burnout, family support, coworker support, and organizational support.

To examine the association between workplace violence exposure and job burnout and the moderating role of social support, three-step hierarchical multiple regression analyses were conducted. In Model 1, workplace violence exposure was entered as the independent variable after controlling for general characteristics, including gender, certification level, number of dispatches, and intention to continue working. In Model 2, family support, coworker support, and organizational support were additionally entered to examine the main effects of each support system. Finally, in Model 3, interaction terms between workplace violence exposure and each social support variable were entered to test whether social support moderated the association between workplace violence exposure and job burnout. Prior to creating the interaction terms, continuous variables were mean-centered to reduce potential multicollinearity.

To assess regression assumptions, the independence of residuals was examined using the Durbin–Watson statistic, and multicollinearity was evaluated using tolerance values and variance inflation factors (VIFs). The observed tolerance values ranged from 0.24 to 0.88 and VIF values ranged from 1.13 to 4.20, indicating no evidence of problematic multicollinearity. A *p*-value of less than 0.05 was considered statistically significant.

### 2.4. Ethical Considerations

Participants were informed about the purpose and procedures of the study, the voluntary nature of participation, their right to refuse or withdraw at any time, and the potential benefits and risks associated with participation. Collected data were used solely for research purposes and stored for three years. Personal identifying information, including contact information collected for the purpose of providing mobile beverage coupons, was immediately destroyed after the completion of the incentive process.

## 3. Results

### 3.1. Demographic and Work-Related Characteristics

A total of 175 paramedics participated in this study. Among them, 131 participants (74.9%) were male and 44 (25.1%) were female. The mean age of the participants was 36.03 ± 5.66 years, with the largest age group being 36 years or older (46.9%). Regarding marital status, 120 participants (68.6%) were married, while 55 (31.4%) were unmarried, divorced, or separated.

In terms of rank, fire sergeants accounted for the largest proportion (31.4%), followed by firefighters (30.9%) and fire lieutenants (30.9%). Most participants were licensed nurses (61.7%), whereas 17.1% were Level 1 Emergency Medical Technicians (EMTs) and 21.1% were Level 2 EMTs. The mean work experience was 6.84 ± 6.71 years, and nearly half of the participants (49.1%) had less than 5 years of work experience.

Regarding the number of dispatches, the majority of participants (60.6%) reported responding to 5–10 dispatches, while 31.4% reported 10–15 dispatches. In addition, 62.3% of the participants indicated an intention to continue working as paramedics. The mean monthly salary was 3.61 ± 0.66 million KRW, and most participants (60.0%) reported earning between 3 and 4 million KRW per month (Table 1).

### 3.2. Descriptive Findings and Correlations of the Study Variables

Descriptive findings and correlations among the study variables are presented in Table 2. The average workplace violence exposure score was 29.43 ± 14.50, while the mean score for job burnout was 42.78 ± 18.39. The mean scores for family support, coworker support, and organizational support were 101.83 ± 21.80, 89.91 ± 19.43, and 46.61 ± 13.16, respectively.

Workplace violence exposure showed a significant positive correlation with job burnout (r = 0.314, *p* < 0.001), indicating that higher levels of workplace violence exposure were associated with higher levels of job burnout. Organizational support showed significant negative correlations with both workplace violence exposure (r = −0.337, *p* < 0.001) and job burnout (r = −0.269, *p* < 0.001). In contrast, family support and coworker support were not significantly correlated with workplace violence exposure or job burnout. Additionally, family support, coworker support, and organizational support were positively correlated with one another.

The range of scores for workplace violence exposure was 16 to 80 (possible range: 16–80), job burnout ranged from 0 to 92 (possible range: 0–96), family support ranged from 27 to 125 (possible range: 25–125), coworker support ranged from 29 to 125 (possible range: 25–125), and organizational support ranged from 15 to 75 (possible range: 15–75).

### 3.3. Factors Influencing Job Burnout: Moderating Effects of Support

The results of the hierarchical regression analysis are presented in Table 3. In Model 1, workplace violence exposure showed a significant positive association with job burnout (β = 0.292, *p* < 0.001), and the model explained 40.3% of the variance in job burnout.

In Model 2, family support, coworker support, and organizational support were additionally entered to examine the main effects of social support variables. Organizational support showed a negative trend with job burnout (β = −0.158, *p* = 0.066), suggesting a trend whereby higher organizational support was associated with lower levels of job burnout. In contrast, family support and coworker support were not significantly associated with job burnout. The explanatory power of the model increased to 41.6%.

**Figure 1:**
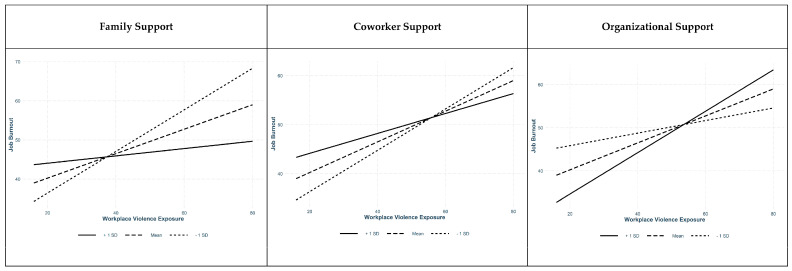
Interaction plots of workplace violence exposure and social support in predicting job burnout. Note: Solid line = high social support (+1 SD); dashed line = mean social support; dotted line = low social support (−1 SD).

In Model 3, interaction terms between workplace violence exposure and social support variables were entered to examine whether social support moderated the association between workplace violence exposure and job burnout. The interaction between workplace violence exposure and family support was statistically significant (β = −0.249, *p* = 0.008), indicating that family support moderated the relationship between workplace violence exposure and job burnout, such that the positive association between workplace violence exposure and burnout became weaker at higher levels of family support. In contrast, the interaction terms for coworker support and organizational support were not statistically significant. The final model explained 47.6% of the variance in job burnout (Adj. R^2^ = 0.434), and the increase in explanatory power from Model 2 to Model 3 was statistically significant (ΔR^2^ = 0.060, *p* < 0.001). To further examine the moderating effect of family support, a simple slope analysis was conducted. The association between workplace violence exposure and job burnout was significant when family support was low (B = 0.532, *p* < 0.001) and at the mean level (B = 0.313, *p* = 0.001), but was no longer significant when family support was high (B = 0.094, *p* = 0.523). These findings further support the buffering role of family support. The interaction plots are presented in Figure 1.

## 4. Discussion

This study examined the association between workplace violence exposure and job burnout among Korean paramedics and explored the role of social support in this relationship. The findings indicated that workplace violence exposure was significantly associated with job burnout, suggesting that higher levels of workplace violence exposure were associated with greater emotional exhaustion and psychological fatigue. These findings are consistent with a previous study involving emergency medical technicians and healthcare workers, which reported workplace violence exposure as a major contributor to occupational stress and burnout [29]. Similar trends have also been reported in international paramedic populations, where traumatic experiences and occupational stress were identified as important contributors to burnout and psychological distress [4,5,6,30].

The present study also found that the relationship between social support and job burnout varied depending on the source of support. Organizational support showed a negative but non-significant association with job burnout in Model 2 (β = −0.158, *p* = 0.066). In other words, higher levels of organizational support were associated with lower levels of job burnout, suggesting that organizational resources and support may play an important role in alleviating the daily occupational stress experienced by paramedics. Organizational support may function not merely as a response to specific incidents, but rather as a structural resource within the overall work environment that may help alleviate cumulative occupational stress associated with emergency response duties. These findings are consistent with previous studies reporting that organizational support was positively associated with job satisfaction and psychological well-being among healthcare workers and public safety personnel [31,32,33]. In particular, a recent international study involving public safety personnel identified organizational culture and supervisory support as key organizational factors affecting mental health and occupational adjustment [33], emphasizing the importance of organizational support systems.

In contrast, family support did not show a direct association with job burnout but was found to buffer the positive association between workplace violence exposure and job burnout. This finding suggests that family support may not be directly associated with lower levels of burnout under ordinary circumstances; however, its role may become more prominent under high-stress conditions, such as exposure to workplace violence. In this context, family support appears to function as a buffering factor that weakens the positive association between workplace violence exposure and job burnout. For paramedics, emotional interactions with family members following traumatic field experiences may facilitate emotional stability and psychological recovery, highlighting the importance of family support as a personal recovery resource. Consistent with these findings, previous studies involving Korean firefighters and international ambulance personnel have reported that family and spouse support play important protective and buffering roles against psychological distress, PTSD symptoms, and maladaptive coping behaviors [34,35,36].

These findings suggest that the functions of social support may differ depending on the source of support. While organizational support was associated with lower levels of job burnout, family support functioned as a situational protective factor operating under stressful conditions. Organizational support may therefore be understood as a structural resource that is associated with occupational well-being, whereas family support may serve as a relational resource that facilitates emotional recovery during crisis situations. These differential roles indicate the need to distinguish the sources and functions of social support rather than treating social support as a single, unified concept. Previous studies involving firefighters and paramedics have highlighted the protective roles of social support and organizational support in post-traumatic adaptation and psychological well-being [19,37,38]. However, relatively few studies have differentiated support sources in detail or examined their distinct functions. By separately examining family, coworker, and organizational support, the present study provides empirical evidence that different sources of support may operate through different mechanisms in relation to job burnout among paramedics. In the present study, coworker support and organizational support did not significantly moderate the association between workplace violence exposure and job burnout. Although these findings suggest that different sources of social support may operate through distinct mechanisms, the reasons underlying these differences remain unclear. Because the present study did not directly examine the processes through which different sources of support influence burnout, caution is warranted when interpreting these findings. Future research should explore the mechanisms underlying source-specific support effects, potentially through qualitative or mixed methods approaches, to better understand how family, coworker, and organizational support function in the context of workplace violence exposure among paramedics. These findings are meaningful because they may inform more targeted intervention strategies; organizational support may be strengthened as a structural resource associated with lower levels of job burnout, whereas family support may serve as a relational resource that buffers the positive association between workplace violence exposure and job burnout. In this respect, the present study extends previous research by demonstrating that different sources of social support may play distinct roles in the context of workplace violence exposure among paramedics. While previous studies have often treated social support as a single construct, the present findings suggest that family support, coworker support, and organizational support may not function in the same manner. These findings contribute to a more nuanced understanding of support mechanisms associated with job burnout among paramedics and provide a foundation for developing source-specific support programs and intervention strategies. Importantly, previous studies have often reported the protective role of social support as a broad construct, providing limited guidance regarding which sources of support should be prioritized in intervention development. By demonstrating that family support, but not coworker or organizational support, moderated the association between workplace violence exposure and burnout, the present study provides evidence that different sources of support may not operate equivalently. This finding suggests that examining social support as a single construct may overlook important source-specific differences in the context of workplace violence exposure among paramedics. Therefore, the present study extends previous research by clarifying the distinct roles of family, coworker, and organizational support and by providing a more practical basis for developing targeted support strategies for paramedics.

From a practical perspective, the present findings highlight the need for multidimensional approaches to prevent job burnout among paramedics. These findings have implications not only for healthcare organizations and emergency medical systems but also for policymakers responsible for occupational health and safety among emergency responders. At the organizational level, efforts should be made to reduce daily occupational stress through institutional support systems, including improvements in the work environment, psychological support programs, and counseling services. Such efforts may be strengthened through the continued implementation and advancement of firefighter mental health support programs currently operated by the National Fire Agency in South Korea. At the same time, considering the importance of family support, it is also necessary to establish support systems that involve family members. For example, educational programs and communication-enhancement interventions targeting both paramedics and their families may help families function as effective support resources.

At the policy level, efforts to prevent workplace violence and strengthen support systems for paramedics should extend beyond incident-specific responses. Policies that promote organizational support, improve access to psychological services, and encourage family involvement in mental health programs may contribute to reducing burnout among paramedics. Given that different sources of support may play distinct roles, support strategies should be designed to address both workplace and family contexts.

This study has several limitations. First, because this was a cross-sectional study conducted among paramedics working in a single region, caution is required when generalizing the findings to all paramedics. In addition, participants were recruited through a voluntary online survey using convenience sampling over a relatively short data collection period, which may have introduced self-selection bias and further limited the representativeness and generalizability of the findings. Second, all variables were measured using self-report questionnaires, which may have introduced subjective response bias. Because all variables were collected from the same respondents using self-report measures, the possibility of common method bias cannot be completely excluded. Furthermore, because of the cross-sectional nature of the study, causal relationships among the variables cannot be established. In addition, perceptions of social support may be influenced by sociocultural factors specific to Korean workplace and family contexts, which were not examined in the present study. Future studies should include larger and more diverse samples from multiple regions and employ longitudinal designs to better examine the temporal relationships among variables. Such approaches would improve the generalizability of the findings and provide a better understanding of the temporal relationships among workplace violence exposure, social support, and job burnout. In addition, further research is needed to examine organizational support factors specifically related to workplace violence situations.

## 5. Conclusions

This study examined the association between workplace violence exposure and job burnout among Korean paramedics and explored the roles of different sources of social support in this relationship. The findings suggest that workplace violence exposure was associated with higher levels of job burnout and that the functions of social support may differ according to its source. In particular, family support moderated the association between workplace violence exposure and job burnout, whereas organizational support was associated with lower levels of job burnout. These findings extend previous research by demonstrating that different sources of social support may not operate in the same manner and highlight the importance of considering source-specific support mechanisms when addressing job burnout among paramedics. By distinguishing family, coworker, and organizational support, the present study contributes to a more nuanced understanding of social support in the context of workplace violence exposure. The findings may inform the development of future intervention strategies, organizational support systems, and family-based support programs aimed at supporting paramedics exposed to workplace violence.

## Figures and Tables

**Table 1:** Demographic and work-related characteristics of the participants (N = 175).

Variables	Characteristics	N (%)	M ± SD
Gender	Male	131 (74.9)	
	Female	44 (25.1)	
Age (years)	≤30	29 (16.6)	36.03 ± 5.66
	31–35	64 (36.6)	
	≥36	82 (46.9)	
Marital Status	Unmarried/Divorced/ Separated	55 (31.4)	
	Married	120 (68.6)	
Rank	Firefighter (Sobangsa)	54 (30.9)	
	Fire Sergeant (Sobanggyo)	55 (31.4)	
	Fire Lieutenant (Sobangjang)	54 (30.9)	
	Fire Captain or higher (Sobangwi)	12 (6.9)	
License	Nurse	108 (61.7)	
	Level 1 Emergency Medical Technician	30 (17.1)	
	Level 2 Emergency Medical Technician	37 (21.1)	
Work Experience (years)	<5	86 (49.1)	6.84 ± 6.71
	5–<10	42 (24)	
	≥10	47 (26.9)	
Number of Dispatches	<5	14 (8)	
	5–<10	106 (60.6)	
	10–15	55 (31.4)	
Intention to Continue Work	Yes	109 (62.3)	
	No	66 (37.7)	
Monthly Salary (KRW)	≤3 million	45 (25.7)	361.54 ± 65.81
	3–4 million	105 (60)	
	>4 million	25 (14.3)	

**Table 2:** Means, Standard Deviations, and Correlations Among Study Variables.

Variables	M	SD	1	2	3	4	5
Workplace violence	29.43	14.50	1				
Burnout	42.78	18.39	0.314 ***	1			
Family support	101.83	21.80	−0.112	−0.039	1		
Coworker support	89.91	19.43	−0.027	−0.070	0.569 ***	1	
Organizational support	46.61	13.16	−0.337 ***	−0.269 ***	0.412 ***	0.608 ***	1

* *p* < 0.05, ** *p* < 0.01, *** *p* < 0.001.

**Table 3:** Factors Influencing Job Burnout: Moderating Effects of Support.

Variables	Model 1			Model 2			Model 3		
	β	t	*p*	β	t	*p*	β	t	*p*
Gender (Ref: Male)									
Female	0.142	2.274	0.024	0.132	2.078	0.039	0.143	2.352	0.020
License (Ref: Nurse)									
Level 1 EMT	−0.126	−2.010	0.046	−0.150	−2.299	0.023	−0.142	−2.179	0.031
Level 2 EMT	−0.304	−4.585	<0.001	−0.309	−4.592	<0.001	−0.333	−5.151	<0.001
Dispatches (Ref: <5)									
5–<10	0.016	0.140	0.889	0.026	0.232	0.817	0.030	0.284	0.776
10–15	−0.047	−0.392	0.695	−0.045	−0.376	0.708	0.054	0.466	0.642
Intention to stay (Ref: Yes)									
No	0.362	5.463	<0.001	0.347	4.883	<0.001	0.275	3.888	<0.001
Workplace violence	0.292	4.669	<0.001	0.248	3.663	<0.001	0.256	3.574	<0.001
Family support				−0.001	−0.018	0.986	0.097	1.207	0.229
Coworker support				0.117	1.315	0.190	0.157	1.815	0.071
Organizational support				−0.158	−1.849	0.066	−0.221	−2.640	0.009
Violence × Family support							−0.249	−2.682	0.008
Violence × Coworker support							−0.091	−0.945	0.346
Violence × Organizational support							0.121	1.679	0.095
R-square	0.403			0.416			0.476		
△R-square	0.403			0.013			0.060		
Adj R-square	0.378			0.380			0.434		
F (*p*)	16.12 (<0.001)			11.68 (<0.001)			11.27 (<0.001)		
△F (*p*)	16.12 (<0.001)			1.19 (0.315)			6.19 (<0.001)		

Durbin-Watson = 2.284, Tolerance = 0.24~0.88, VIF = 1.13~4.20.

## Data Availability

The datasets generated and/or analyzed during the current study are not publicly available due to privacy and ethical restrictions. De-identified data may, however, be made available from the corresponding author on reasonable request and with Institutional Review Board approval.
